# Environmental, Ecological and Food Resources in the Biodiversity Overview: Health Benefits

**DOI:** 10.3390/life11111228

**Published:** 2021-11-13

**Authors:** Alessandra Durazzo, Massimo Lucarini

**Affiliations:** CREA-Research Centre for Food and Nutrition, Via Ardeatina 546, 00178 Rome, Italy

## 1. Introduction

The Special Issue “Environmental, Ecological and Food Resources in the Biodiversity Overview: Health Benefits” wants to underline the importance of classification, cataloguing and analysis of environmental, agricultural, ecological, botanical and food sources—from native species to unconventional sources and wastes—which should be promoted from the perspectives of biodiversity and sustainability [1]. In this sense, studies on botanical classification and on the optimization of the diversity of species should be encouraged, and monitoring the biochemistry of wild species, intra-species biodiversity or environmental influences and their effects on food qualities and health benefits become key issues [2,3]. For instance, Zeleke et al. [2] described the land-use impact on stand structure and fruit yield of *Tamarindus indica* L. in the drylands of southeastern Ethiopia; although the majority of farmland trees produced <5000 fruit year^−1^, the selection of Tamarind germplasm in its natural ranges could improve production [2]. Another example is given by Durazzo et al., [3] that showed a representation of biodiversity, sustainability, and health of bee products.

Studies on the impact of biodiverse environments in food production and nutrition should be promoted, especially in terms of the exploitation and promotion of wild and native foods from the perspective of responsible human consumption and health outcomes.

Management, modelling and the sharing of environment and biodiversity information and data are encouraged. Addressing to the promotion of diversities, to building synergies throughout food, agriculture, sustainability, nutrition and health systems and sharing of knowledge represent a future integrate and multidisciplinary direction, in the perspective of one health outcomes [1,4].

The production of food composition data for nutrients and biologically active compounds, as well as the definition of new biomarkers and indicators, represent key determinants in understanding the linkage and crosslinks between food, environment, nutrition and health [5,6,7,8,9]. In this integrated dimension, studies on mapping the diversity of food products, food systems and consumer preferences should be promoted [10,11,12]

The Special issue explored the developments, management, applications and utilizations of agricultural, ecological, botanical, food and environmental interoperable, multidisciplinary and multi-purpose resources, repositories and infrastructures from environmental to nutritional- and health- related fields. Current research trends are towards integrated web-based models, e-services and e-platforms in the perspective of data sharing and digital era.
